# Identification and experimental validation of cuproptosis regulatory program in a sepsis immune microenvironment through a combination of single-cell and bulk RNA sequencing

**DOI:** 10.3389/fimmu.2024.1336839

**Published:** 2024-06-14

**Authors:** Tingru Zhao, Yan Guo, Jin Li

**Affiliations:** Department of Clinical Laboratory, Key Clinical Laboratory of Henan Province, The First Affiliated Hospital of Zhengzhou University, Zhengzhou, Henan, China

**Keywords:** sepsis, cuproptosis, ScRNA-seq, bulk RNA-seq, LST1

## Abstract

**Background:**

In spite of its high mortality rate and poor prognosis, the pathogenesis of sepsis is still incompletely understood. This study established a cuproptosis-based risk model to diagnose and predict the risk of sepsis. In addition, the cuproptosis-related genes were identified for targeted therapy.

**Methods:**

Single-cell sequencing analyses were used to characterize the cuproptosis activity score (CuAS) and intercellular communications in sepsis. Differential cuproptosis-related genes (CRGs) were identified in conjunction with single-cell and bulk RNA sequencing. LASSO and Cox regression analyses were employed to develop a risk model. Three external cohorts were conducted to assess the model’s accuracy. Differences in immune infiltration, immune cell subtypes, pathway enrichment, and the expression of immunomodulators were further evaluated in distinct groups. Finally, various *in-vitro* experiments, such as flow cytometry, Western blot, and ELISA, were used to explore the role of LST1 in sepsis.

**Results:**

ScRNA-seq analysis demonstrated that CuAS was highly enriched in monocytes and was closely related to the poor prognosis of sepsis patients. Patients with higher CuAS exhibited prominent strength and numbers of cell–cell interactions. A total of five CRGs were identified based on the LASSO and Cox regression analyses, and a CRG-based risk model was established. The lower riskScore cohort exhibited enhanced immune cell infiltration, elevated immune scores, and increased expression of immune modulators, indicating the activation of an antibacterial response. Ultimately, *in-vitro* experiments demonstrated that LST1, a key gene in the risk model, was enhanced in the macrophage in response to LPS, which was closely related to the decrease of macrophage survival rate, the enhancement of apoptosis and oxidative stress injury, and the imbalance of the M1/M2 phenotype.

**Conclusions:**

This study constructed a cuproptosis-related risk model to accurately predict the prognosis of sepsis. We further characterized the cuproptosis-related gene LST1 to provide a theoretical framework for sepsis therapy.

## Background

Sepsis refers to a life-threatening dysfunction of organs caused by an imbalanced host response to infection, leading to an uncontrolled syndrome of inflammation and immune dysregulation resulting in organelle dysfunction. This condition is strongly linked to elevated levels of illness and mortality (1). When infected, the Toll-like receptors bind to pathogen-associated molecular patterns, thereby provoking the secretion of proinflammatory cytokines that initiate an inflammatory reaction (2, 3). A cytokine storm arises when the inflammatory response becomes unmanageable, eventually leading to microcirculatory disorders and multiple organ failure. Unfortunately, managing sepsis has yielded limited advancement (4). Traditionally, PCT and CRP have been widely utilized for diagnosing and predicting sepsis, although their effectiveness remains restricted. Recently, data-driven approaches have been employed to enhance the characterization, early detection, classification, prognosis, and treatment of sepsis (5). Thus, the identification of novel biomarkers is urgently necessary to enable an early diagnosis, evaluate the response to early treatment, and predict the prognosis of sepsis patients.

In recent studies, cuproptosis has emerged as a novel form of copper-dependent cell death, distinct from apoptosis, ferroptosis, or necroptosis (6, 7). Intracellular accumulation of copper can lead to oxidative stress and impair cellular function, while copper homeostasis is carefully regulated (8, 9). The buildup of copper can result in the binding of lipoylated enzymes in the tricarboxylic acid cycle, triggering protein aggregation, proteotoxic stress, and ultimately cell death (10). Researchers have identified several cuproptosis-related genes (CRGs) that contribute to disease progression, with a primary focus on tumor advancement, which may impact the tumor immune microenvironment and predict the effectiveness of immunotherapy (11–14). Additionally, studies have indicated that CRG signatures are closely associated with certain inflammation-related conditions, including inflammatory bowel disease (15), ulcerative colitis (16, 17), and Alzheimer’s disease (18, 19). Considering that sepsis is characterized by a systemic inflammatory response, we hypothesize that cuproptosis may play a role in sepsis. CRGs might be involved in its early diagnosis, treatment, and prognosis.

Over the past decade, single-cell sequencing has emerged as a powerful technology in next-generation sequencing, encompassing single-cell DNA sequencing, single-cell RNA sequencing, and single-cell epigenomic sequencing (20). Single-cell sequencing has gained popularity in sepsis research, leading to numerous studies exploring the transcriptomic features and immunological landscape of sepsis (21, 22). While the characteristics of different single-cell subsets in sepsis have been discussed (23, 24), little is known about the impact of immunological genomic signatures on regulating sepsis. To date, there have been no studies explicitly focusing on CRG signatures in septic patients, and the comprehensive characteristics of CRGs and their relationship with the inflammatory response and prognosis of septic patients remain unclear.

In this investigation, we acquired scRNA-seq data and bulk RNA-seq data of septic samples from the GEO database to explore the regulation patterns of cuproptosis comprehensively. We exhibited the immune landscape of septic patients and performed further analysis to ascertain the association between the activity score of cuproptosis [referred to as cuproptosis activity score (CuAS)] and the immune microenvironment. Utilizing the WGCNA algorithm, LASSO, and Cox regression analysis, we identified the prognostic CRGs and used them to develop a riskScore consisting of five genes. To validate the predictive accuracy of the riskScore, we employed three additional independent GEO datasets. Furthermore, we constructed a novel nomogram that integrates the riskScore with clinicopathological characteristics to estimate the clinical application of riskScore features in sepsis prognosis. Additionally, we evaluated the disparities in enriched pathways and immunological characteristics between distinct riskScore groups in sepsis patients. Ultimately, through *in-vitro* experiments, we investigated the role of LST1, a crucial gene associated with cuproptosis, in promoting sepsis-induced injury.

## Materials

### Acquisition of raw data

We obtained peripheral blood single-cell data from two normal individuals and five hospitalized patients diagnosed with gram-negative bacterial sepsis at 0 and 6 h after diagnosis (25) from the GEO website (GSE167363). We also downloaded the bulk sepsis transcriptome data (GSE65682, GSE95233, GSE63042, GSE106878) consisting of 802, 129, and 124 blood samples and 94 circulating leukocyte samples from sepsis patients, respectively. GSE65682 was selected as the training set, and after excluding samples with missing prognostic information, a total of 42 normal, 114 sepsis non-survivors, and 365 sepsis survivors were yielded in GSE65682. To ensure data quality, we performed log2 transformation and normalization using the robust multiple array average (RMA) function in the “affy” R package. We extracted a total of 41 cuproptosis-related genes (CRGs) based on previous studies (26).

### scRNA-seq data processing and cell annotation

scRNA-seq data were processed using the “Seurat” R package. We filtered out genes expressed in less than five single cells and cells with fewer than 200 or more than 4,000 genes. Additionally, we removed cells with over 25% mitochondrial genes to retain high-quality scRNA-seq data. After these filtering steps, we identified a total of 59,695 suitable cells. Next, we applied the “NormalizeData” and “ScaleData” techniques to normalize and scale the remaining cells. To identify highly variable genes for further analysis, we used the “FindVariableFeatures” function and identified the top 2,000 hypervariable genes. We employed the “RunHarmony” function to mitigate batch effects since the data originated from multiple samples. We used principal component analysis (PCA) for dimensionality reduction and identified anchor points. We used the t-distributed stochastic neighbor embedding (t-SNE) algorithm to discover meaningful clusters and tested the top 15 principal components. Subsequently, we identified 12 cell clusters using the “FindNeighbors” and “FindClusters” functions with a resolution of 0.3. Finally, we visualized these clusters using a “t-SNE” diagram. Cell-type annotation was performed using the “Celltypist” Python package by previous findings (27).

### Cuproptosis activity score

The CuAS for each cell lineage was determined using the single-sample gene set enrichment analysis (ssGSEA) and “UCell” algorithms from the “irGSEA” R package. The cells were then classified into high- and low-CuAS groups based on the 75% quantile values of the ssGSEA score.

### Cell communication analysis

Cell communication analysis was conducted using the “CellChat” R package based on the CellChatDB.human ligand–receptor interaction database. CellChat objects were created for each group (low and high CuAS) based on the UMI count matrix (28) for each group (low and high CuAS). The default parameters were used for the analysis. The CellChat objects from each group were combined using the “mergeCellChat” function to compare the total number and strength of interactions. Differences in the number and strength of interactions among distinct cell types between groups were visualized using the “netVisual_diffInteraction” function. Signaling pathways with differential expression were determined using the “identifyOverExpressedGenes” function. The distribution of signaling gene expression between groups was visualized using the “subsetCommunication” and “netVisual_chord_gene” functions.

### ssGSEA and WGCNA analysis

The ssGSEA algorithm was utilized to calculate the percentage of absolute enrichment of a specific gene set in each sample. In this particular investigation, we implemented the ssGSEA method to assign cuproptosis enrichment values to each individual in the GSE65682 dataset.

A signed weighted co-expression network of GSE65682 was constructed utilizing the “WGCNA” package available in R software (29). The detailed procedures were conducted as follows: First, we eliminated any genes with missing values using the “goodSamplesGenes” function. Subsequently, we visually determined the optimal soft threshold (softPower = 9) for adjacency computation. To reveal the interconnectedness of the network, we converted the expression matrix into an adjacency matrix, which was further transformed into a topological overlap matrix (TOM). Utilizing the variations in TOM, we performed average linkage hierarchical clustering. Then, to integrate modules exhibiting high correlation coefficients, we dynamically pruned the hierarchical clustering tree to identify similar modules with a minimum module size set to 60. The module eigengenes (MEs), representing all genes within a specific module, served as the main component for further analysis. Pearson correlation analysis was applied to explore the relationship between eigengene values and clinical characteristics. Eventually, we selected module genes that exhibited the most remarkable correlation with CuAS for in-depth analysis.

### Construction and validation of the riskScore

Univariable analysis was conducted to determine genes significantly correlated with patient survival. We then used the least absolute shrinkage and selection operator (LASSO) Cox regression analysis to identify genes closely associated with prognosis and calculate the corresponding risk coefficients, as previously reported (30–32). In addition, we performed statistical analysis of survival using log-rank tests to identify the gene combination with the smallest *p*-value, which was considered the final characteristic gene. To assess the predictive ability of our findings, we calculated a risk score for each sepsis patient based on the coefficients identified from the log-rank tests. Patients were then categorized into high- and low-risk groups based on the median risk score. We used the Kaplan–Meier method to plot survival curves based on these risk groups.

Additionally, we employed receiver operating characteristic (ROC) curves to evaluate the efficacy of our prediction model. An area under the curve (AUC) value greater than 0.7 indicated excellent performance. The predictive ability of the signature was validated in three independent GEO datasets utilizing AUC.

### Assessment of the prognostic model

To provide a more comprehensive prognostic model, we developed a nomogram that combines riskScore with age and gender as independent prognostic factors. We used calibration curves to evaluate the accuracy of the nomogram in predicting the probability of overall survival at 28 days. Furthermore, we used decision curve analysis (DCA) to assess the net benefit of the nomogram in comparison to clinical characteristics alone.

### Enrichment analysis

Gene ontology enrichment analysis was carried out using the “clusterProfiler” R package (33), based on the Kyoto Encyclopedia of Genes and Genomes (KEGG) and Gene Ontology (GO). The biological functions examined included biological processes (BP), molecular functions (MF), and cellular components (CC). Statistical significance was determined for *p*-values below 0.05. To assess heterogeneity in biological processes and pathway activities, gene set variation analysis (GSVA) enrichment was conducted using the “GSVA” R package (34). The preferred gene sets for GSVA were selected from the Molecular Signatures Database (MSigDB) under the hallmark gene set “c5.go.bp.v7.5.1.symbols.” Various molecular features were discovered within DDR subtypes. Differences in biological functions and signaling pathways were calculated using the “limma” R package, with absolute t-values and a GSEA score above 2 considered statistically significant. Additionally, pathway activities were examined through gene set enrichment analysis (GSEA) using the “clusterProfiler” R package. Normalized enrichment scores (NES) were ranked, and statistical significance was determined for *p*-values below 0.05. Activity scores for classical disease-related signaling pathways were calculated between groups using the progeny R package, with *p*-values below 0.05 considered statistically significant.

### Sepsis immunity

The immune-infiltrating levels were assessed using ssGSEA algorithms, which evaluated the proportions of different immune cells in each sample based on global marker genes. The fractional enrichment or relative abundance of each immune cell subset was calculated using the algorithms. Differences in immune infiltration levels between groups were determined using the Wilcoxon rank-sum test. A heatmap was generated using different algorithms to visually display the abundance of immune infiltration in each sepsis sample. The “ESTIMATE” R package was used to estimate the immune infiltration levels in sepsis patients. Immune checkpoints consist of various molecules expressed on immune cells that regulate the level of immune activation. These molecules, including antigens, cell adhesion molecules, co-inhibitors, co-stimulators, ligands, and receptors, play an essential role in limiting excessive immune activation. We compared the expression levels of well-known immune checkpoint genes in both groups.

### Preparation and culture of macrophages

RAW264.7 cells, a mouse macrophage cell line, were purchased from Beyotime Biotechnology (Shanghai, China) and cultured in complete medium consisting of high-glucose DMEM (Gibco, NY, United States), 10% fetal bovine serum (FBS, Gibco, NY, United States), penicillin (100 U/mL), and streptomycin (100 mg/mL) at 37°C with 5% CO_2_. The cells were then exposed to LPS (100 ng/mL) for 24 h to mimic a septic injury model.

### RNA extraction and RT-PCR

The total RNA of macrophages was extracted utilizing the TRIzol reagent (Invitrogen Life Technologies, NY, United States). Subsequently, the cDNA was synthesized utilizing the PrimeScript RT reagent (TaKaRa, Otsu, Japan) following the manufacturer’s instructions. Quantitative PCR reactions were conducted using a Power SYBR Green PCR master mix (Applied Biosystems, CA, United States) in the Applied Biosystems 7500 sequence detection system. Relative quantification of mRNA expression was calculated using the 2^−ΔΔCt^ method. The expression level of each mRNA was calculated by the standard curve method and normalized with GAPDH. All samples for each mRNA were run in triplicate and independently repeated four times. Primers used for PCR analyses are detailed in **Supplementary Table S1**.

### Cell viability and cytotoxicity assay

RAW264.7 cells were incubated with or without LPS for 24 h in a 96-well plate. CCK8 solution (Beyotime Biotechnology, Shanghai, China) was added to each well and incubated for 1 h, and the OD value at 450 nm was measured to determine cell viability. The cytotoxicity of the cells was evaluated using the LDH Cytotoxicity Assay Kit (Beyotime Biotechnology, Shanghai, China), following the manufacturer’s instructions. The supernatants were collected, and the OD value at 490 nm was measured to determine LDH activity. The data were presented as folds of LDH release compared to control cells.

### Western blot assay

Cell lysates were collected from RAW264.7 cells using the western cell lysis buffer (Beyotime Biotechnology, Shanghai, China). The total protein was quantified with a Pierce BCA Protein Assay Kit (Thermo Fisher Scientific, MA, United States). The PVDF membrane was incubated with a primary antibody against LST1 (Abcam, Cambridge, United Kingdom, 1:2,000), followed by incubation with the secondary antibody, goat against mice (ABclonal, MA, United States, 1:5,000). Protein bands were visualized using a WB imaging instrument (Bio-Rad, CA, United States) after using an enhanced ECL immunoblotting detection reagent (Thermo Fisher Scientific, MA, United States). Densitometric analysis of the Western blot results was performed using the ImageJ software.

### Flow cytometry analysis

RAW264.7 cells were incubated in 6-well plates and exposed to various treatments. The cells were digested in 0.25% trypsin (without EDTA), centrifuged at 300×*g* for 3 min, and then resuspended in 300 μL of binding buffer. Subsequently, the cell suspension was incubated with LST1 (LMAI Bio, Shanghai, China) for 30 min on ice to assess the expression of LST1. In addition, we also analyzed the subset of macrophages by staining the RAW264.7 cells with antibodies against iNOS (M1 marker, eBioscience, CA, United States) and CD206 (M2 marker, eBioscience, CA, United States) for 30 min on ice. The fluorescence signal was analyzed using a FACScalibur cytometer (BD Biosciences, CA, United States), and the data were processed using FlowJo 10.0 version software.

### shRNA knockdown

The shRNA plasmids targeting LST1 and negative control shRNA were purchased from GeneChem (Shanghai, China) for a knockdown. The indicated shRNA lentiviral virus was packed in 293 T cells. RAW264.7 cells were transfected with lentivirus and selected using 2 μg/mL of puromycin for 72 h.

### Detection of oxidative stress-related markers

MDA, SOD, and GSH-Px detection kit (Beyotime Biotechnology, Shanghai, China) was used to measure the oxidative stress level of RAW264.7 cells following the manufacturer’s guidelines.

### Statistical analysis

We analyzed statistics using different software programs, namely, R 4.1.0, GraphPad Prism 8.0, and SPSS 22.0. In order to compare the survival rates of the two groups, we utilized Kaplan–Meier curves along with a log-rank test. All survival curves were generated using the “ggsurvplot” R package. To assess prognostic variables, we employed univariable Cox regression analysis. Moreover, we utilized LASSO regression to identify the factors that had a more significant impact on sepsis outcomes. The R software package “ggplot2” was employed to visualize the data, while the R package “survival” was used to calculate the overall survival (OS). To examine the relationship between two continuous variables, we performed Spearman’s correlation analysis. To compare the difference in continuous variables between the two groups, we employed either the Wilcoxon sum-rank test or the two-tailed *t*-test. Furthermore, we used the chi-square test to make comparisons regarding non-continuous variables between the two groups. In cases where there were three or more groups, one-way ANOVA was employed to assess differences. Prior to conducting the analysis, we checked the normality of data distribution using the Kolmogorov–Smirnov test and evaluated the homogeneity of variances using Levene’s test. A statistically significant value was considered as *p <*0.05.

## Results

### The scRNA profiling of non-sepsis donors and septic patients

**Figure 1:**
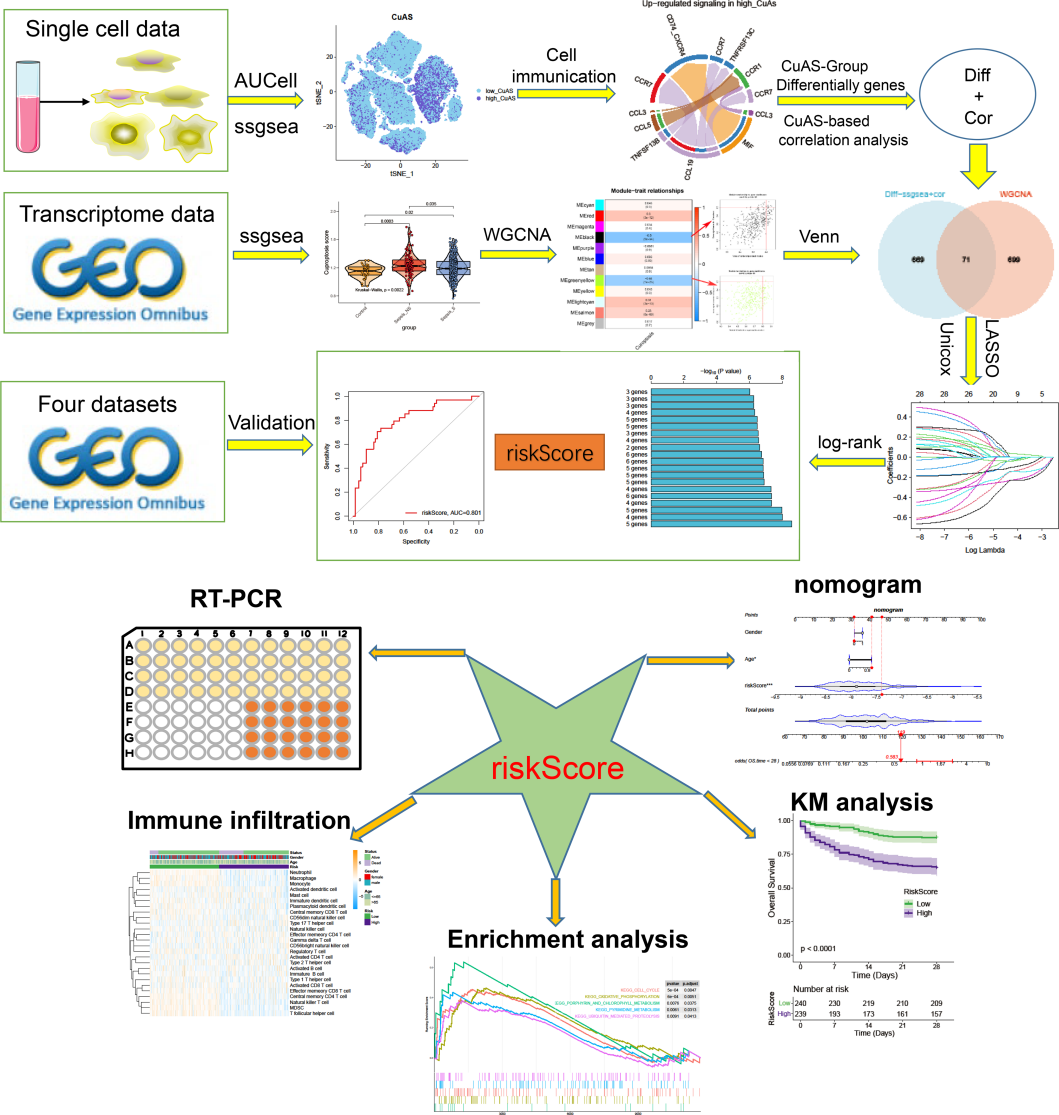
The flowchart of this study.

**Figure 2:**
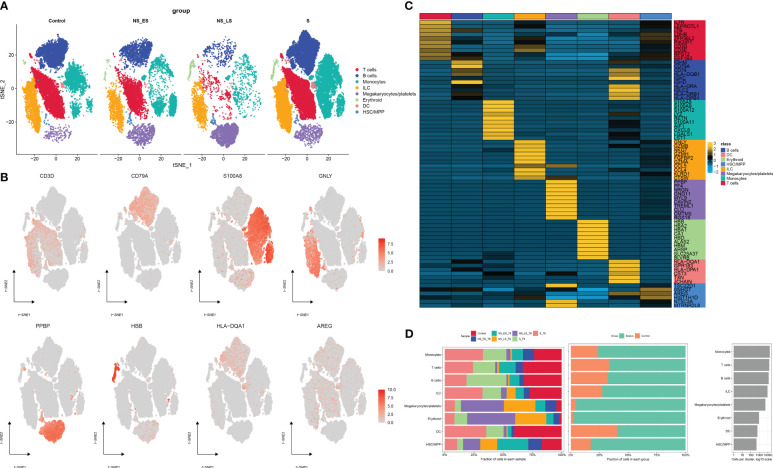
Annotation of single-cell data. **(A)** The t-SNE plots demonstrate the presence of all cell categories in healthy donors, as well as in early-stage non-survived septic patients (NS_ES), late-stage non-survived septic patients (NS_LS), and survived septic patients (S). **(B)** Representative marker genes for each cell group are shown. **(C)** A heatmap illustrates the expression patterns of the top marker genes for each cell subtype. **(D)** The distribution of each cell type is compared between healthy donors and septic patients.

**Figure 1** presents the flowchart depicting the methodology employed in this research. To comprehensively examine the global landscape of cuproptosis, we initially utilized one single-cell dataset, known as GSE167363, which consisted of two control samples and 10 septic samples derived from five individuals suffering from sepsis. This dataset was further subjected to analysis after undergoing quality assessment based on cell signatures and the proportion of mitochondrial genes. Subsequently, a total of 59,695 cells of high quality (43,902 cells from septic patients and 15,793 cells from healthy controls) were deemed suitable for further investigation. The distribution of cells within each sample was predominantly consistent, as observed in **Supplementary Figure S1A**, thereby allowing for subsequent exploration. The application of t-SNE, a method of dimensionality reduction, facilitated the classification of all cells into 12 distinct clusters, which were subsequently categorized as T cells, B cells, monocytes, ILC, megakaryocyte/platelets, erythroid, DC, and HSC/MPP, as depicted in **Supplementary Figures S1A, B**. The representation in **Figure 2A** illustrates the distribution of cell categories across healthy donors, the early stage of non-surviving septic patients (NS_ES), the late stage of non-surviving septic patients (NS_LS), and surviving septic patients (S). **Figure 2B** serves as an illustration of typical markers associated with different cell types. Additionally, **Figure 2C** displays a heatmap showcasing the expression patterns of the top 10 marker genes specific to each cell subtype. The inclusion of samples from various groups resulted in the differentiation of cell categories within the sepsis and control groups, highlighting the enrichment of all cell subtypes, primarily in sepsis samples, as demonstrated in **Figure 2D**.

### Analysis of cuproptosis activity score

**Figure 3:**
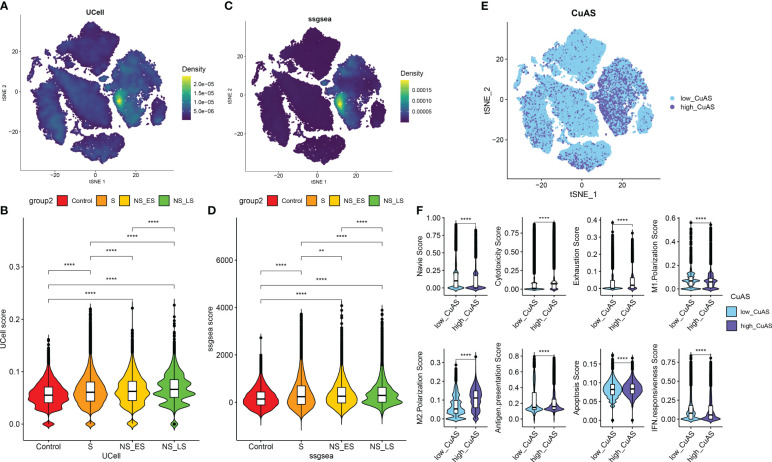
Characteristic of cuproptosis activity score (CuAS) at the single-cell level. **(A)** Analysis of CuAS based on UCell algorithm scores reveals significant differences. **(B)** Comparison of UCell scores between the control and septic groups. **(C)** Evaluation of CuAS using the ssGSEA algorithm scores shows distinct patterns. **(D)** Differences in ssGSEA scores between the control and septic groups are observed. **(E)** The distribution of high-CuAS cells and low-CuAS cells is shown. **(F)** Naive, cytotoxicity, exhaustion, M1 polarization, M2 polarization, antigen presentation, apoptosis, and IFN responsiveness scores are compared between high-CuAS cells and low-CuAS cells. **p < 0.01, ****p < 0.0001.

We then utilized the “ssGSEA” and “UCell” algorithms to compute the CuAS of every cell subtype. Notably, higher CuAS values were predominantly observed in various cell types, with a significant concentration in monocytes (**Figures 3A, C**). Our findings also indicated a notable elevation in CuAS among septic patients, particularly in those in the later stages and non-survivors (**Figures 3B, D**). In addition, we established an *in-vitro* sepsis model and examined the expression of 10 critical CRGs using RT-PCR analysis. The results showed that LPS insult could increase the levels of DLD, SLC31A1, ABCB6, ATOX1, CYP1A1, and MTF1 but decreased the levels of FDX1 and LIPT1. Interestingly, the levels of CDKN2A and ATP7A remained unchanged after LPS damage (**Supplementary Figure S2**). These results suggest a strong association between cuproptosis and an unfavorable prognosis in sepsis patients.

Subsequently, we categorized single cells from septic patients into high- and low-CuAS groups based on the 75th percentile values of the ssGSEA score (**Figure 3E**). A total of 10,975 single cells were classified as the high-CuAS group, while the remaining 32,927 cells were classified as the low-CuAS group. According to our investigation, cells exhibiting high CuAS demonstrated enhanced levels of cytotoxicity, exhaustion, M2 polarization, antigen presentation, and apoptosis scores, whereas cells with low CuAS displayed stronger native, M1 polarization, and IFN responsiveness activity (**Figure 3F**).

### Cell–cell interactions within low-CuAS and high-CuAS cells

**Figure 4:**
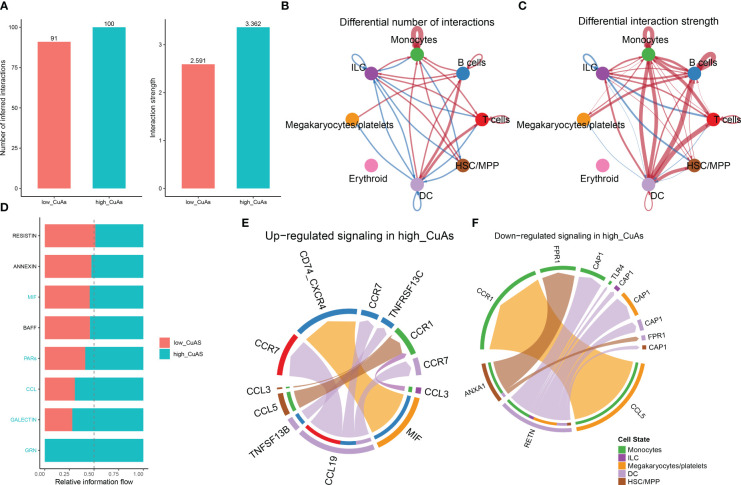
Intercellular communication difference between high-CuAS cells and low-CuAS cells. **(A)** Bar charts highlight the disparities in interaction numbers and strengths in the cell–cell communication network between high-CuAS and low-CuAS cells. **(B, C)** Thicker lines indicate stronger interactions, while red or blue colors denote increased or decreased signaling in low-CuAS patients. **(D)** Stacked plots depict the variations in intercellular signaling pathways between high-CuAS and low-CuAS patients. **(E, F)** The chord diagram reveals frequent upregulation or downregulation of signal communications between cell states.

Subsequently, to ascertain the correlation between cuproptosis with immune cells, CellChat analysis was performed to elucidate differences in cell–cell interactions between low- and high-CuAS groups. The numbers and strength of these interactions increased as we transitioned from low CuAS to high CuAS (**Figure 4A**). Notably, the high-CuAS group displayed more robust interaction numbers and strengths with other cell types, including monocyte, T cells, B cells, DC, and HSC/MMP cells, compared to the low-CuAS group (**Figures 4B, C**). By comparing the interaction strengths of each pathway, we identified specific pathways that were more active in the high-CuAS cells than the low-CuAS cells. Notably, signaling pathways such as MIF, PARs, CCL, GALECTIN, and GRN showed significant activation in high-CuAS cells (**Figure 4D**). To illustrate, the upregulation of the CCL3/5 and CCR1 signaling pathways in high-CuAS cells primarily manifested as increased communication between HSC/MPP (senders) and monocytes (receivers). Additionally, frequent communication between HSC/MPP (senders), monocyte (receivers), and DC (receivers) led to impaired ANXA1 and FPR1 signaling pathways in high-CuAS cells (**Figures 4E, F**).

### Identification of the most relevant genes for cuproptosis

**Figure 5:**
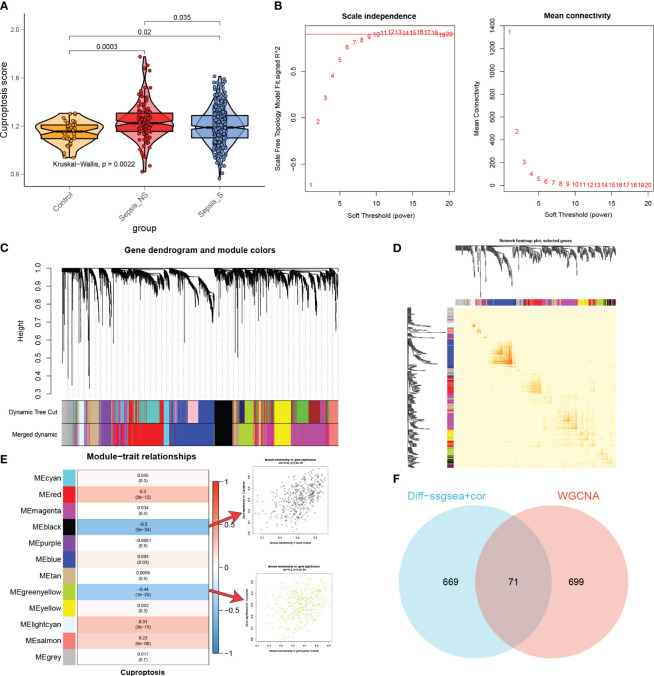
The most relevant genes for cuproptosis. **(A)** A comparison was made between the CuAS in sepsis patients and healthy donors. **(B)** The scale independence and mean connectivity of various soft threshold values (*β*) were analyzed. The red numbers indicate different soft threshold values (1–20), while the red lines highlight the selected cutoff values. **(C)** Clustering dendrograms, along with their assigned module colors, were constructed based on the dissimilarities among all genes. **(D)** A network heatmap plot was generated, representing the topological overlap matrix of all analyzed genes. **(E)** WGCNA analysis was performed to identify the modules most significantly associated with cuproptosis, showing the relationship between significant module membership and gene significance. **(F)** Venn plots were used to identify the genes strongly associated with cuproptosis.

The ssGSEA algorithm was performed to determine the absolute enrichment percentage of cuproptosis enrichment values in individuals in the GSE65682 dataset. As shown in **Figure 5A**, in sepsis patients, CuAS levels were significantly higher compared to healthy donors, with non-survived patients exhibiting greater CuAS levels than survived patients. To investigate the gene co-expression network in the sepsis cohort, we constructed the WGCNA network using the expression profile data of sepsis. The optimal soft threshold *β* was set to 9, as shown in **Figure 5B**. Subsequently, we detected gene modules based on variations in TOM and identified 12 gene modules in this analysis, as shown in **Figures 5C–E**. The black and green-yellow modules exhibited the highest correlation with sepsis, as depicted in **Figure 5E**. We obtained 770 specific genes related to cuproptosis within these two modules for further enrichment analyses. GO enrichment analysis revealed that cuproptosis-associated genes were enriched in processes such as neutrophil degranulation, immune effector process regulation, leukocyte-mediated immunity, and immune receptor activity (**Supplementary Figure S3A**). By performing KEGG enrichment analysis, we found that cuproptosis-associated genes were enriched in hematopoietic cell lineage, glutathione metabolism, *Staphylococcus aureus* infection, and viral protein interaction with cytokine and cytokine receptor (**Supplementary Figure S3B**).

Moreover, we conducted differential analyses to identify CuAS-related DEGs between high- and low-CuAS groups, resulting in the selection of 730 DEGs for further investigation. Additionally, correlation analysis was performed to identify genes most closely associated with CuAS, and we selected the top 150 genes for subsequent analysis. The DEGs and the genes obtained from the correlation analysis were then merged, resulting in a total of 740 genes that were found to have the most significant impact on CuAS in the single-cell analysis. Finally, we intersected these 740 CRGs with the two modular genes most associated with cuproptosis obtained from WGCNA, leading to the selection of 71 overlapped genes for further analysis (**Figure 5F**).

### Identification of prognosis genes associated with sepsis and construction of a risk model

**Figure 6:**
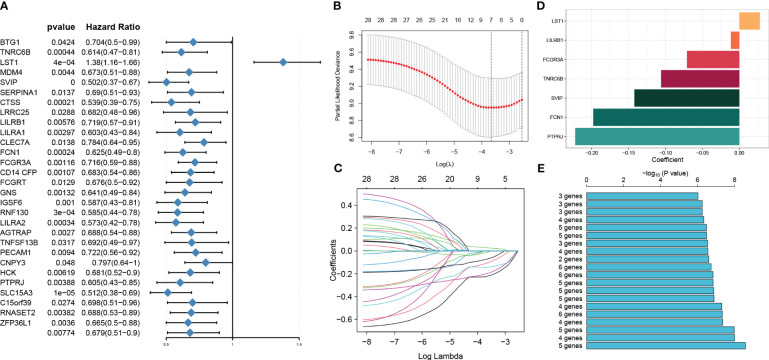
Identification of prognosis genes associated with sepsis and construction of a risk model. **(A)** Through univariable analysis, 30 genes were found to correlate with patients’ overall survival statistically. **(B, C)** LASSO Cox regression analysis was then conducted to filter further for prognosis genes with non-zero coefficients. **(D)** The specific coefficient values of the seven genes associated with cuproptosis were obtained using the LASSO algorithm and the optimal lambda value. **(E)** The CuAS signatures and their performance in predicting the prognosis of sepsis were evaluated.

We conducted a univariable Cox regression analysis on 71 overlapping genes to identify genes that exhibit a statistically significant correlation with patient survival (*p* < 0.05). This analysis identified 29 genes as protective factors and one gene as a risk factor (**Figure 6A**). To refine our selection and focus specifically on prognosis genes, we performed LASSO Cox regression analysis, which resulted in the identification of seven genes with non-zero coefficients (**Figures 6B–D**). These genes include small VCP/p97-interacting protein (SVIP), leukocyte immunoglobulin-like receptor, subfamily B member 1 (LILRB1), Fc fragment of IgG, low-affinity IIIa, receptor (CD16a) (FCGR3A), trinucleotide repeat containing 6B (TNRC6B), leukocyte-specific transcript 1 (LST1), collagen/fibrinogen domain-containing protein 1 (FCN1), and protein tyrosine phosphatase receptor type J (PTPRJ). These seven genes yielded a total of 127 gene combinations. Subsequently, we calculated the survival curve and *p*-value for each combination using the log-rank (Mantel–Cox) test. Interestingly, the combination of a five-gene signature exhibited the lowest *p*-value, suggesting its better predictive ability for sepsis prognosis (**Figure 6E**). Therefore, based on the coefficient value and expression corresponding to each gene, we construct a riskScore using the following formula: riskScore = (−0.2274916 × PTPRJ expression) + (−0.2093238 × FCN1 expression) + (−0.1423479 × SVIP expression) + (−0.0223379 × LILRB1 expression) + (0.0316062 × LST1 expression). K-M analysis reveals the impact of each member of the riskScore model on overall survival in sepsis patients (**Supplementary Figure S4**).

### Validation of riskScore and clinicopathological parameters

**Figure 7:**
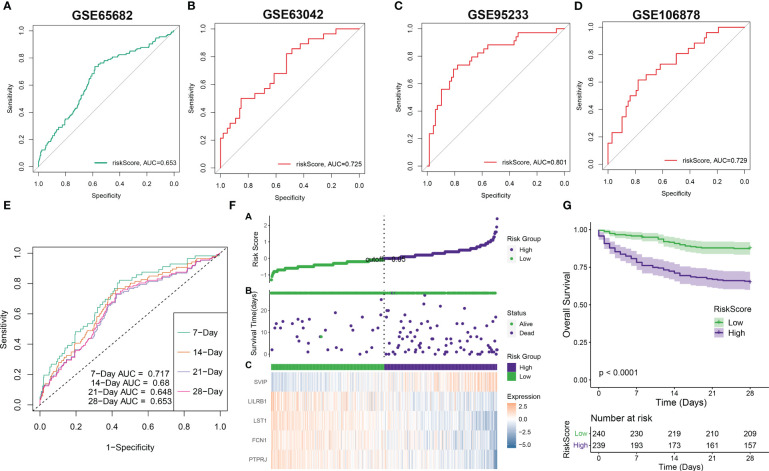
Validation of riskScore in independent datasets. **(A–D)** The ROC curve values of the five-gene signature riskScore in four separate datasets (GSE65682, GSE63042, GSE95233, and GSE106878) were determined. **(E)** The ROC curve value of the five-gene signature riskScore was analyzed for different stages of sepsis in the GSE65682 dataset. **(F)** The riskScore distribution in sepsis patients and the correlation between riskScore and survival data were represented in scatter plots. High-risk and deceased patients were denoted by purple dots, while green dots denoted low-risk and surviving patients. A heatmap displayed the expressions of the five-gene signature in septic patients with varying riskScores. **(G)** The overall survival times of high-risk and low-risk groups were compared using a Kaplan–Meier survival curve.

Subsequently, we employed ROC curves to evaluate the performance of the predictive model in different datasets (GSE65682, GSE63042, GSE95233, and GSE106878). The AUC values obtained from all four datasets were as follows: AUC = 0.653 (GSE65682), 0.725 (GSE63042), 0.801 (GSE95233), and 0.729 (GSE106878) (**Figures 7A–D**), indicating that the constructed riskScore can predict the prognosis of sepsis to some extent. In the GSE65682 dataset, the AUCs for predicting the 7-, 14-, 21-, and 28-day OS were 0.717, 0.68, 0.648, and 0.653, respectively (**Figure 7E**). Next, based on the median, we categorized septic patients in the GSE65682 dataset into high- and low-riskScore groups. Survival analysis revealed a markedly worse OS in septic patients with high risk (**Figures 7F, G**).

**Figure 8:**
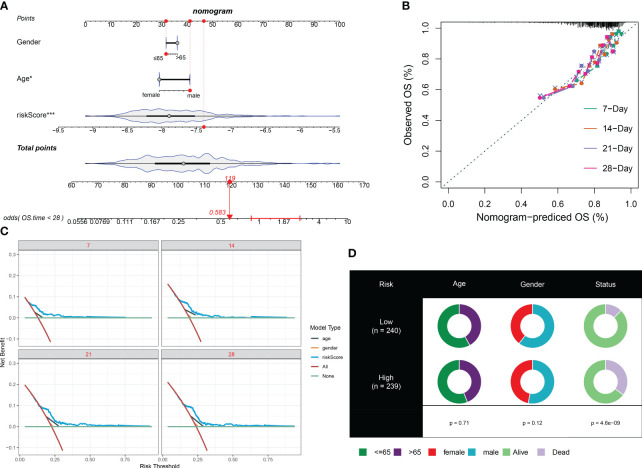
Prediction accuracy of CuAS and clinicopathological parameters. **(A)** A nomogram model was constructed to integrate the riskScore and clinicopathological parameters. **(B)** Calibration curves were generated to assess the accuracy of the nomogram model for predicting outcomes at 7, 14, 21, and 28 years. **(C)**. A decision curve was plotted to evaluate the clinical usefulness of the nomogram model at 7, 14, 21, and 28 years. **(D)** The proportion of clinicopathological parameters was analyzed in septic patients with high or low riskScore.

To enhance the accuracy of outcome prediction in septic patients, we developed predictive nomograms that incorporated riskScores and clinic pathological features (age and sex) using the GSE65682 dataset (**Figure 8A**). The calibration plot, utilizing clinical outcome parameters at 7, 14, 21, and 28 days, demonstrated the nomogram’s effectiveness in accurately forecasting survival outcomes (**Figures 8B–D**).

### Characterization of pathway enrichment between risk groups

**Figure 9:**
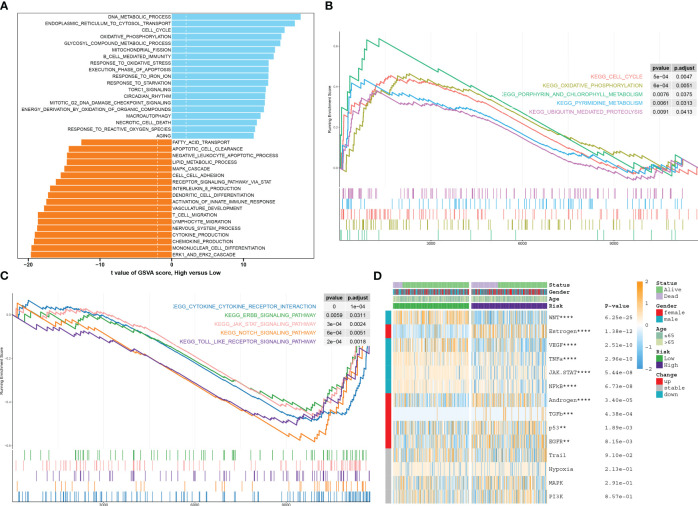
Characterization of pathway enrichment between risk groups. **(A)** The *t*-value of the GSVA scores was used to rank the differences in biological functions between high-risk and low-risk septic patients. **(B, C)** The GSEA scores of signaling pathways revealed the upregulated and downregulated main pathways between high-risk and low-risk septic patients. **(D)** A heatmap displayed the expression profiles of 13 characteristic CuAS between low-risk and high-risk septic patients. Patient annotations included status, age, gender, and risk subtypes.

Analysis of function annotations using the GSVA algorithm revealed that high-risk subtypes primarily exhibited biological functions associated with the ERK1/2 cascade, differentiation of mononuclear cells, production of chemokines, processes in the nervous system, migration of lymphocytes and T cells, and development of the vasculature. On the other hand, low-risk subtypes showed a strong association with biological functions such as DNA metabolic processes, transport of substances from the endoplasmic reticulum to the cytosol, the cell cycle, oxidative phosphorylation, metabolic processes involving glycosyl compounds, fission of mitochondria, B-cell-mediated immunity, and response to oxidative stress (**Figure 9A**). Similarly, results from GSEA revealed that the pathways upregulated in high-risk subtypes included the cell cycle, oxidative phosphorylation, metabolism of porphyrin and chlorophyll, pyrimidine metabolism, and ubiquitin-mediated proteolysis (**Figure 9B**). Conversely, the pathways upregulated in low-risk subtypes included cytokine–cytokine receptor interaction, ERBB, JAK-STAT, NOTCH, and Toll-like receptor signaling pathways (**Figure 9C**). The heatmap displayed distinct expression patterns of 13 well-known disease-related signaling pathways between high-risk and low-risk patients, with five pathways found to be significantly upregulated in high-risk patients and five pathways showing enhanced activity in low-risk patients (**Figure 9D**).

### Differences in immunity and therapeutic between distinct RiskScore patients

**Figure 10:**
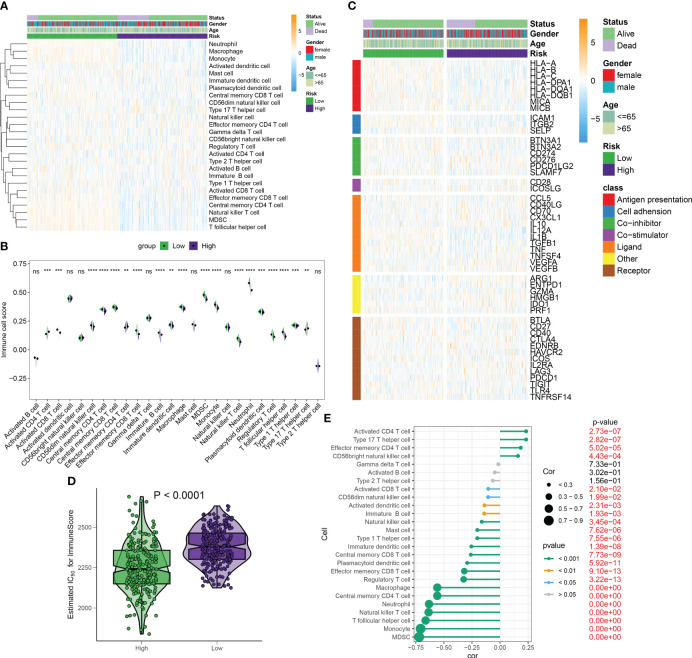
Differences in immunity and therapeutic between distinct RiskScore patients. **(A)** The heatmap visualized the landscape of immune infiltration cells for each septic sample under the ssGSEA algorithm. **(B)** The Wilcoxon rank-sum test results showed infiltration levels of multiple immune cell subtypes in high- and low-risk septic patients. **(C)** The heatmap visualized the landscape of immune modulators and immune checkpoints between septic patients with different risk scores. **(D)** ImmuneScore in septic patients with different risk scores. **(E)** Correlation between riskScore and immune cell subtypes. **p < 0.01, ***p < 0.001, ****p < 0.0001, ns, no significance.

Under the ssGSEA algorithm, the immune infiltration cells in each septic sample were visualized using a heatmap in **Figure 10A**. The analysis of the Wilcoxon rank-sum test results revealed higher levels of infiltration for various immune cell subtypes in the high-RiskScore subtypes. These subtypes included type 17 T helper cells, activated CD4 T cells, and effector memory CD4 T cells. Conversely, the low-RiskScore subtypes showed the presence of activated CD8 T cells, effector memory CD8 T cells, neutrophils, natural killer T cells, regulatory T cells, MDSCs, and monocytes (**Figure 10B**). Additionally, there were significant differences in immune modulators and immune checkpoints between septic patients with different RiskScores. Notably, immune genes associated with antigen presentation (HLA-A, HLA-B, HLA-C, HLA-DPA1, HLA-DQB1, MICA, and MICB), cell adhesion (ICAM1 and ITGB2), co-inhibitors (BTN3A1, BTN3A2, CD274, and SLAMF7), ligands (CCL5, IL12A, IL1B, TGFB1, and TNF), receptors (CD27, CD40, HAVCR2, TIGIT, and TNFRSF14), and other immune modulators (PRF1) were significantly higher in patients with lower riskScores. Conversely, patients with higher riskScores exhibited lower levels of cell adhesion (SELP), ligands (CX3CL1 and IL10), receptors (EDNRB, IL2RA, and PDCD1), and other immune modulators (ARG1, ENTPD1, and HMGB1) (**Figure 10C**; **Supplementary Figure S5**). The observation of a weakened immune score in septic patients with higher riskScores indicates poor responsiveness to a bacterial-induced inflammatory response (**Figure 10D**). Correlation analysis further demonstrated that a higher riskScore was negatively correlated with most immune cell types, suggesting a weaker inflammatory response (**Figure 10E**).

### Validation of the expression of LST1 in LPS-induced macrophages

**Figure 11:**
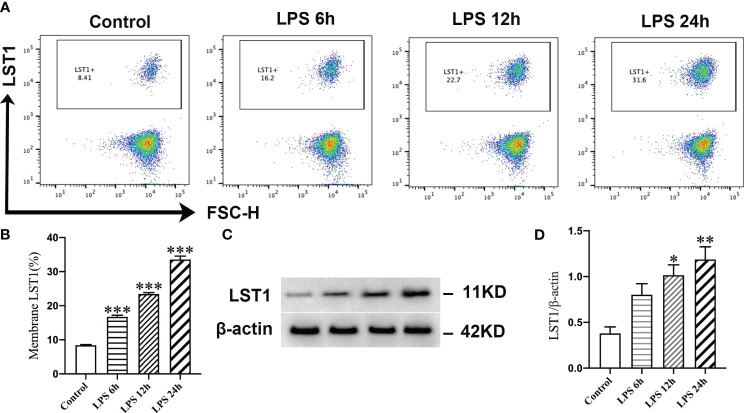
LPS promoted the expression of LST1 in macrophages. **(A)** Flow cytometry analysis of LST1 expression in RAW264.7 cells. **(B)** The percentage of LST-positive macrophage is shown (*n* = 3). **(C)** Represent the Western blot bands of LST1 and β-actin. **(D)** Quantification of protein expression of LST1 (*n* = 3). *p < 0.05, **p < 0.01, ***p < 0.001 versus the control group.

Out of the five CRGs examined in this study, the association between septic injury and LST1 has yet to be investigated. We first looked at the expression levels of LST1 in each cell type of single-cell samples, and the result showed that LST1 was mainly enriched in monocytes (**Supplementary Figure S6**). Subsequently, we focused our research on uncovering the role of LST1 in sepsis through a series of experimental procedures. Initially, the expression of LST1 in RAW264.7 cells was assessed using flow cytometry and Western blot analysis. The evaluation of LST-positive cell proportions revealed a significant increase in LST1 expression in macrophages at 6 h post-LPS stimulation compared to controls. This enhanced expression of LST1 persisted for up to 24 h following LPS-induced injury (**Figures 11A, B**). Moreover, the levels of LST1 protein in macrophages displayed an initial rise at 6 h and gradually increased from 12 to 24 h after LPS exposure (**Figures 11C, D**). These results indicate a time-dependent alteration in LST1 expression in macrophages within the LPS-induced injury model. Taken collectively, these findings suggest that LST1 potentially plays a role in the pathophysiology of sepsis.

### Inhibition of LST1 protects against LPS-induced macrophage damage

**Figure 12:**
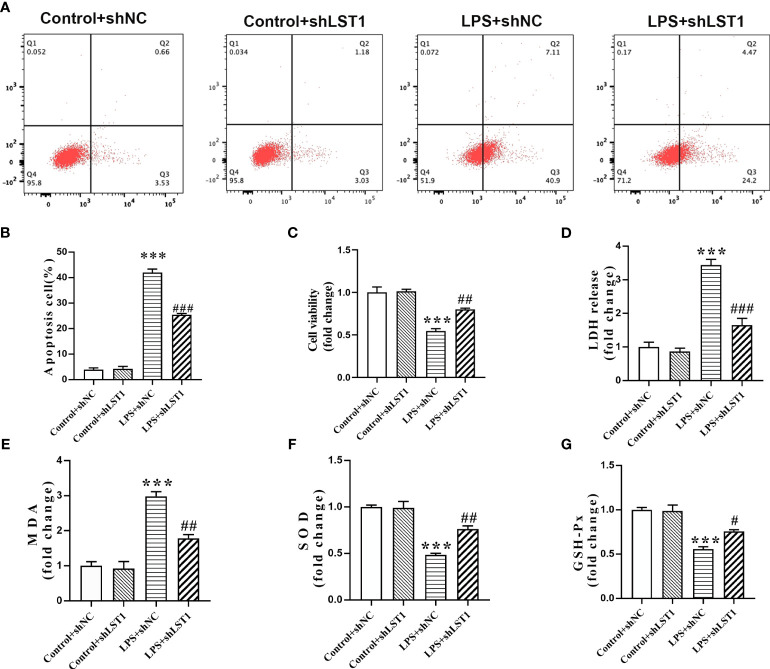
Inhibition of LST1 protects against LPS-induced macrophage damage. **(A)** Apoptotic level was analyzed by flow cytometry after LPS stimulation for 24 h **(B)** The percentage of apoptotic RAW264.7 cells is shown (*n* = 3). **(C)** Cell viability was measured by CCK8 analysis after LPS stimulation for 24 h **(D)** Cytotoxicity was measured by LDH release analysis after LPS stimulation for 24 h **(E–G)** Oxidative stress was evaluated by detection of MDA, SOD, and GSH-Px in RAW264.7 cells after LPS stimulation for 24 h. ***p< 0.001 versus control + shNC group, ^#^ *p* < 0.05, ^##^ *p* < 0.01, ^###^ *p* < 0.001 versus the LPS group.

To determine the impact of inhibiting LST1 on septic injury, we transfected lentivirus-shLST1 into RAW264.7 cells and used flow cytometry to analyze the level of apoptosis among different groups. As expected, the increased rate of apoptosis induced by LPS was reduced in the group where LST1 was knocked down (**Figures 12A, B**). To further validate the effects of LST1 on cell viability and toxicity following sepsis damage, we conducted the analysis using CCK8 and LDH release in the RAW264.7 cells where LST1 was inhibited. We observed a significant increase in cell viability and a marked decrease in toxicity (**Figures 12C, D**). Additionally, we evaluated the effects of LST1 on the activation of oxidative stress in RAW264.7 cells, both in the absence and presence of LPS insult. No significant difference in oxidative stress markers was observed between LV-shNC- and LV-shLST1-transfected RAW264.7 cells when not stimulated with LPS. However, in the LST1-inhibited RAW264.7 cells after LPS stimulation, the peroxide product MDA was significantly reduced, and the antioxidant products SOD and GSH were markedly elevated (**Figures 12E–G**).

**Figure 13:**
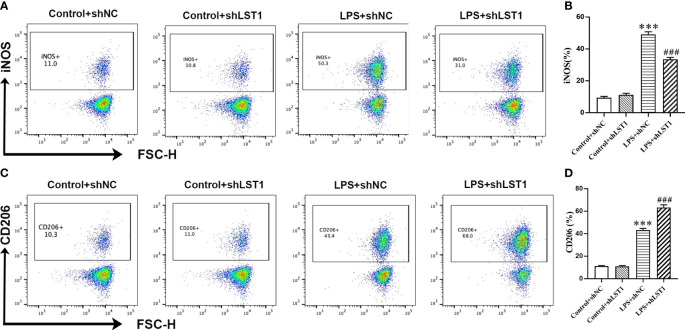
Inhibition of LST1 switches macrophage M1 phenotype to M2 phenotype. **(A)** iNOS expression on macrophages was analyzed by flow cytometry. **(B)** The percentage of iNOS-positive macrophage is shown (*n* = 3). **(C)** CD206 expression on macrophages was analyzed by flow cytometry. **(D)** The percentage of CD206-positive macrophage is shown (*n* = 3). ***p < 0.001 versus control+shNC group, ^###^ *p* < 0.001 versus the LPS group.

To assess the impact of inhibiting LST1 on the M1/M2 phenotypic balance of macrophages in sepsis, we stimulated RAW264.7 cells with LPS to induce the M1 phenotype. We observed a decrease in the percentage of iNOS-positive macrophages (M1 markers) and an increase in the percentage of CD206-positive macrophages (M2 markers) in the RAW264.7 cells treated with LST1 inhibition compared to those treated with LV empty (**Figures 13A–D**). These findings suggest that inhibiting LST1 could improve the proinflammatory and oxidative stress status and protect macrophages from apoptosis.

## Discussion

The connection between sepsis and septic shock and the high mortality rates, ranging from 30% to 60%, remains persistent (35). Furthermore, sepsis continues to be the leading cause of critical care deaths (36). Early detection, diagnosis, and rational therapeutic interventions can effectively manage sepsis and reduce its mortality rate. However, due to the lack of specific biomarkers, the diagnosis and treatment of sepsis remain challenging (37). To address this, the utilization of scRNA-seq provides a valuable approach to detect immune cell alterations associated with sepsis resulting from bacterial pneumonia in a dynamic disease progression. Teng et al. (21) examined peripheral blood mononuclear cells (PBMCs) obtained from healthy controls and two sepsis patients. They observed an upregulated interferon-gamma response, activation and exhaustion properties in T/NK cell subtypes, increased NK cell-induced plasma cells, and activation of T/NK cell subtypes driven by IL-1β signaling pathways. Recently, monocytes have garnered increased attention in understanding the development of sepsis, as the presence of monocyte aggregates in sepsis is linked to a poor clinical outcome (38). In our current study, a total of 12 distinct cell clusters were identified from the analysis of two control samples and 10 septic samples using scRNA-seq. We found that the proportion of monocytes was significantly higher in survivors of sepsis than in normal individuals, suggesting that they primarily mediate immune inflammatory responses in the early stages of sepsis. Consistently, in a multicenter international European prospective study (39), monocyte distribution width (MDW) emerged as a superior early indicator of sepsis in emergency departments compared to PCT or CRP. However, the proportion of monocytes in septic deaths was even lower than in normal subjects, suggesting that monocytes became immunosuppressed as sepsis progressed, ultimately exacerbating disease damage. Several recent studies have focused on constructing prognosis models for sepsis using scRNA-seq and RNA-seq data (40–42). Cuproptosis, a form of mitochondrial cell death triggered by copper, has been linked to immune infiltration in tumors and autoimmune diseases (14). Song et al. discovered a significant correlation between sepsis-induced cardiomyopathy and cuproptosis (43). However, it remains unclear whether cuproptosis genes influence the development and prognosis of sepsis. In our current study, we calculated the CuAS to determine the association between cuproptosis and sepsis prognosis based on the UCell or ssGSEA algorithms. We found that CuAS gradually increased as sepsis progressed and was mainly enriched in a part of monocytes. In addition, the high-CuAS group exhibited enhanced cytotoxicity and immune exhaustion activity. Combined with the reduced proportion of monocytes in non-survivors of sepsis, we hypothesized that cuproptosis may lead to immune exhaustion of monocytes in the late stage of sepsis, ultimately resulting in disease exacerbation. Therefore, the constructed CuAS may indicate the severity and outcome of sepsis, reflecting the status of the immunosuppressive function.

After performing an intersection of scRNA-seq data with two modular genes obtained from WGCNA analysis, we identified a total of 71 common differentially expressed genes (CRGs). These CRGs were then utilized to construct a novel prognostic prediction model. Using a combination of LASSO and Cox regression analysis, we identified a five-gene signature (SVIP, LILRB1, LST1, FCN1, and PTPRJ) as the most compelling prognostic indicator for septic patients. Among the identified prognosis genes, LST1 stood out as the only poor prognostic factor. It was found that LST1, a multifunctional gene encoded within the MHC class III region, exhibited enhanced expression in response to lipopolysaccharides, interferon-gamma, and bacterial stimuli (44). SVIP was a critical regulator of endoplasmic reticulum-associated degradation (ERAD), specifically through its involvement in the transfer of very low-density lipoprotein (VLDL) (45). On the other hand, LILRB1, a receptor expressed on immune cells, binds to MHC class I molecules on antigen-presenting cells. It is believed to play a crucial role in controlling inflammatory responses and cytotoxicity, thereby aiding in the modulation of immune responses and limiting autoreactivity (44). FCN1 encodes ficolin 1, an essential precursor for the complement lectin pathway. Ficolin family proteins are pattern-recognition molecules that interact with sugars present on microbial surfaces. They have been closely associated with circulating phagocytes and have been implicated in the pathogenesis of early-onset sepsis (46). PTPRJ, a protein tyrosine phosphatase (PTP) family member, is expressed in all hematopoietic lineages. It has been shown to negatively regulate T-cell receptor (TCR) signaling (47). All five genes in our risk signature may collectively contribute to the modulation of the inflammatory response associated with sepsis. To evaluate the predictive performance of our risk signature, we constructed ROC curves, nomograms, and calibration curves and conducted DCA. Our results demonstrated that the riskScore, derived from our riskScore model, can predict the prognosis of sepsis patients to some extent. Importantly, our riskScore exhibited higher accuracy in predicting short-term prognosis in septic patients.

Using the constructed riskScore, we categorized sepsis patients into two groups based on their low- and high-risk levels. Through pathway enrichment analysis, it was revealed that patients with higher riskScores exhibited lower levels of ImmuneScore and a decreased infiltration of immune cells compared to patients with lower riskScores. Previous studies indicated that the majority of patients experiencing prolonged sepsis display an immunosuppressive state, with late-stage sepsis-related immunosuppression being a significant contributor to mortality in these patients (23). Upon analyzing the distribution of immune cell subtypes, we discovered that patients with a lower risk score possess a higher abundance of innate immune cells and cytotoxic T cells, highlighting their more excellent antibacterial activity. The low-risk score group also suggests an intensified activation of pathways involved in negative leukocyte apoptotic processes, the activation of the innate immune response, and cytokine production. It is worth noting that this increased activation of pathways was positively correlated with an enhanced antibacterial immune response. Furthermore, when assessing typical immunomodulatory factors and immune checkpoints, it was observed that patients with a low-risk score showcased an upregulation of antigen presentation molecules. This finding suggests that the early activation of the innate immune system and efficient antigen presentation play a pivotal role in preventing sepsis-induced death in patients.

As a constituent member of the identified CRGs, the role of LST1 in sepsis has not been fully elucidated. Previous studies have indicated that LST1 is primarily expressed in immune cells, with elevated levels of LST1 observed in various inflammatory diseases, such as viral infections, rheumatoid arthritis, and inflammatory bowel disease (48, 49). In our current investigation, we observed an increase in LST1 expression in macrophages over time in a model of LPS-induced injury, indicating its close association with the progression of sepsis. Interestingly, the knockdown of LST1 resulted in increased cell viability, reduced cell apoptosis and toxicity, improved resistance against oxidative stress insults, and maintained a balanced M1/M2 macrophage phenotype. These findings strongly suggest that LST1 plays an extensive role in sepsis-induced damage.

There are two areas for improvement in the current research. Firstly, our study did not elucidate the potential mechanism of LST1 in promoting septic insult. Secondly, we only validated the role of LST1 in sepsis *in vitro*, and further validation is needed at the *in-vivo* level.

## Conclusions

We described the cuproptosis level of cells in sepsis at the single-cell level. Based on the regulators of cuproptosis, we constructed a cuproptosis-related risk model to predict the early diagnosis of sepsis accurately. In addition, LST1, a cuproptosis-related molecule in the model, was proven to be enhanced in the macrophage in response to LPS. Finally, we found that LST1 regulated the M1/M2 polarizing phenotypic transformation of macrophages and aggravated sepsis-induced cell injury.

## Data availability statement

The datasets presented in this study can be found in online repositories. The names of the repository/repositories and accession number(s) can be found in the article/**Supplementary Material**.

## Ethics statement

The animal study was approved by the Institutional Animal Care and Use Committee of The First Affiliated Hospital of Zhengzhou University. The study was conducted in accordance with the local legislation and institutional requirements.

## Author contributions

TZ: Methodology, Supervision, Writing – review & editing. YG: Data curation, Methodology, Writing – original draft. JL: Data curation, Methodology, Visualization, Writing – original draft.
