# Supplementary material for: Identification and experimental validation of cuproptosis regulatory program in a sepsis immune microenvironment through a combination of single-cell and bulk RNA sequencing
Source: Front Immunol. 2024 Jun 14;15:1336839. doi: 10.3389/fimmu.2024.1336839 (PMC11211538; doi:10.3389/fimmu.2024.1336839)
Supplement: Supplementary file 1 [file DataSheet_1.docx]

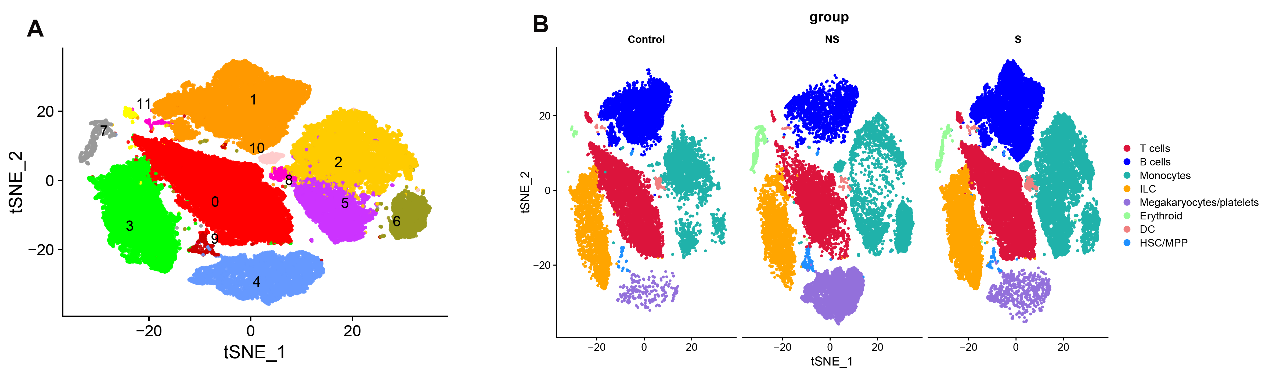


**Supplementary Figure 1**

**A.** The t-SNE plot showed that all the cells in 12 clusters. **B.** Annotated subtypes of 12 cell clusters in healthy donors, survived septic patients and non-survived patients.


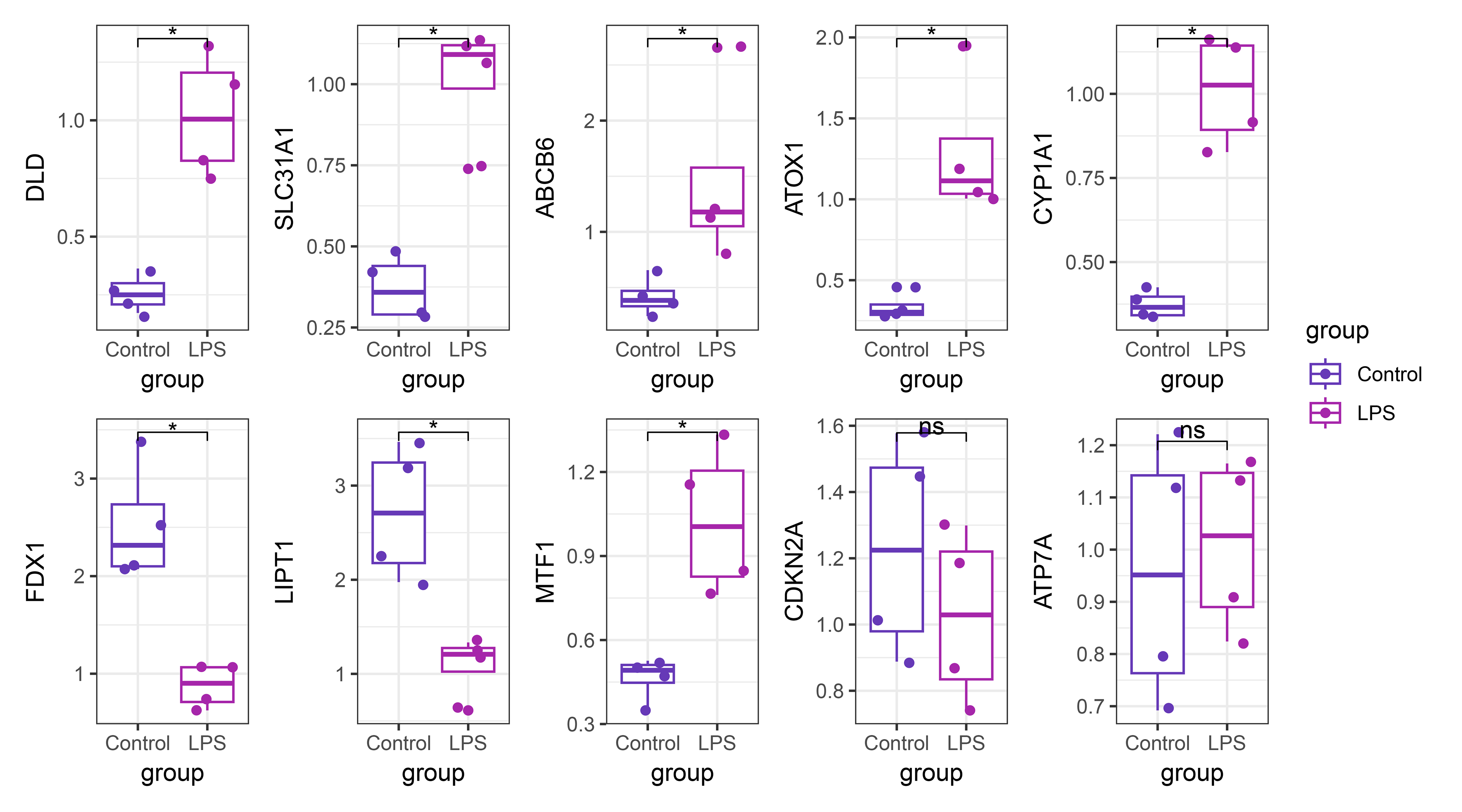


**Supplementary Figure 2**

RT-PCR analysis of 10 critical CRGs in sepsis model in vitro


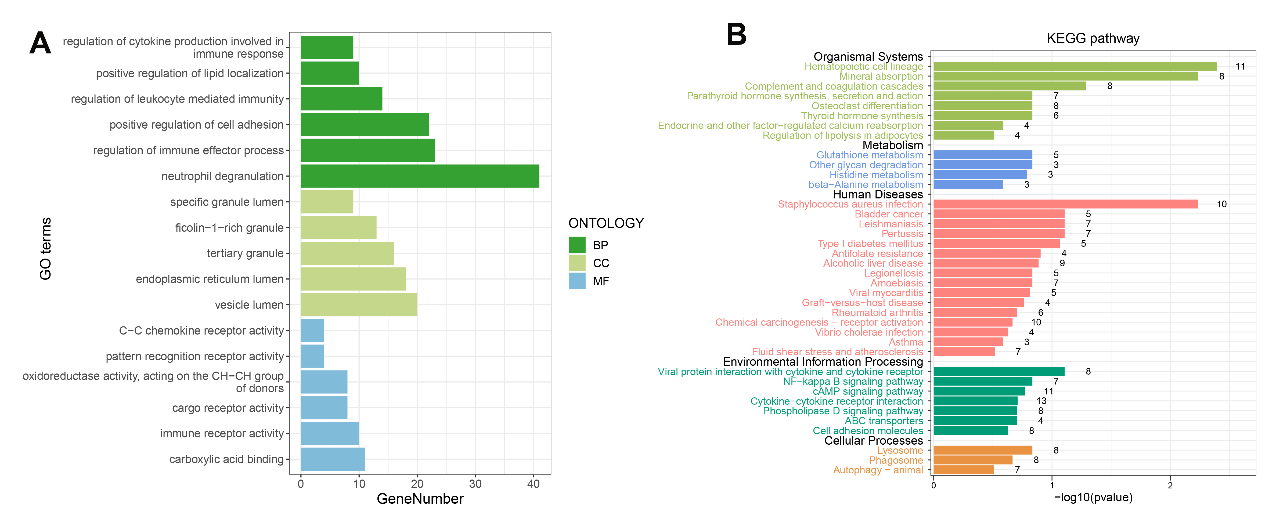


**Supplementary Figure 3**

**A-B.** GO and KEGG enrichment analyses of cuproptosis associated genes.


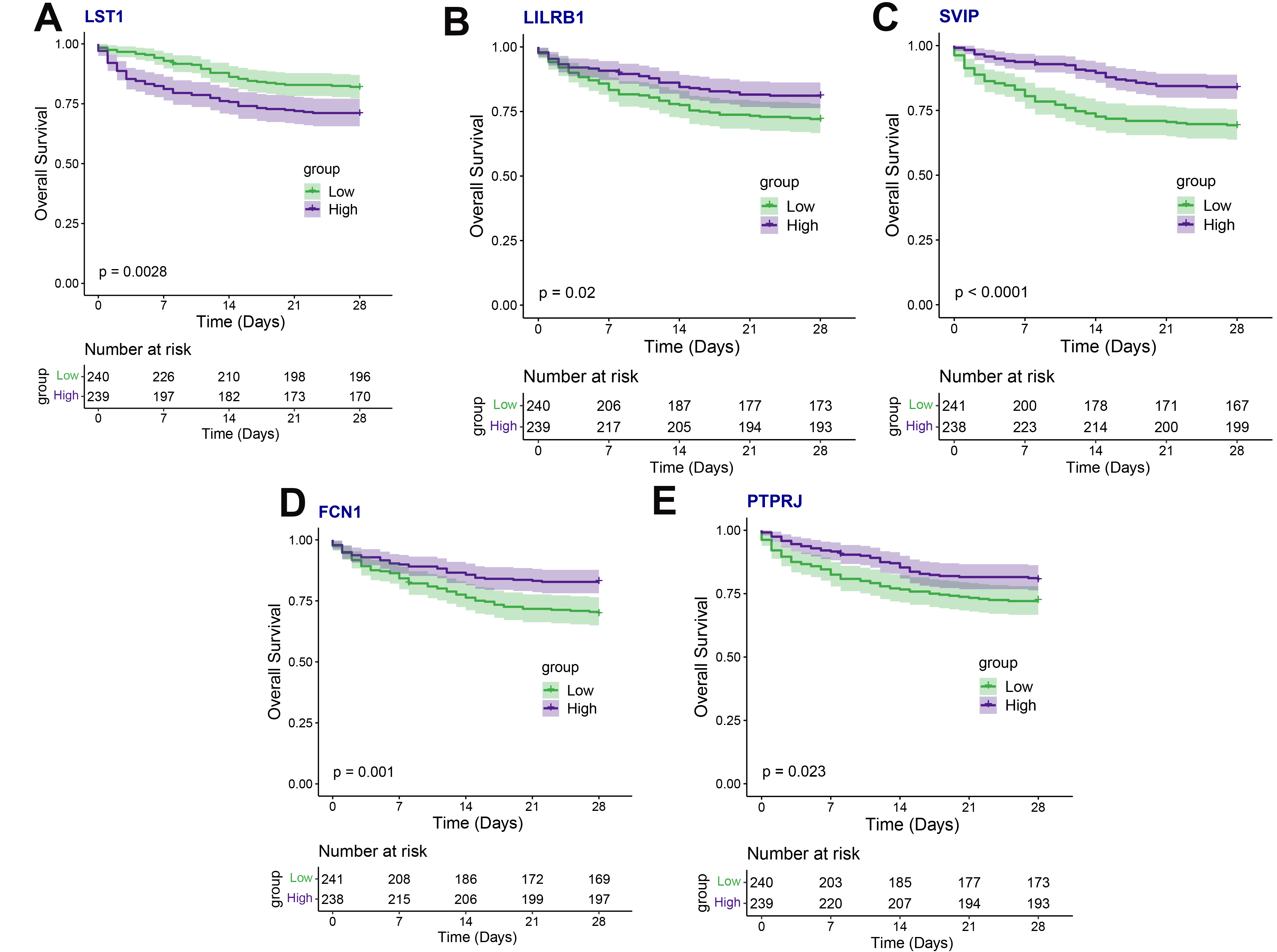


**Supplementary Figure 4**

**A-E.** K-M analyses of 5 genes in riskScore model in the overall survival of septic patients.


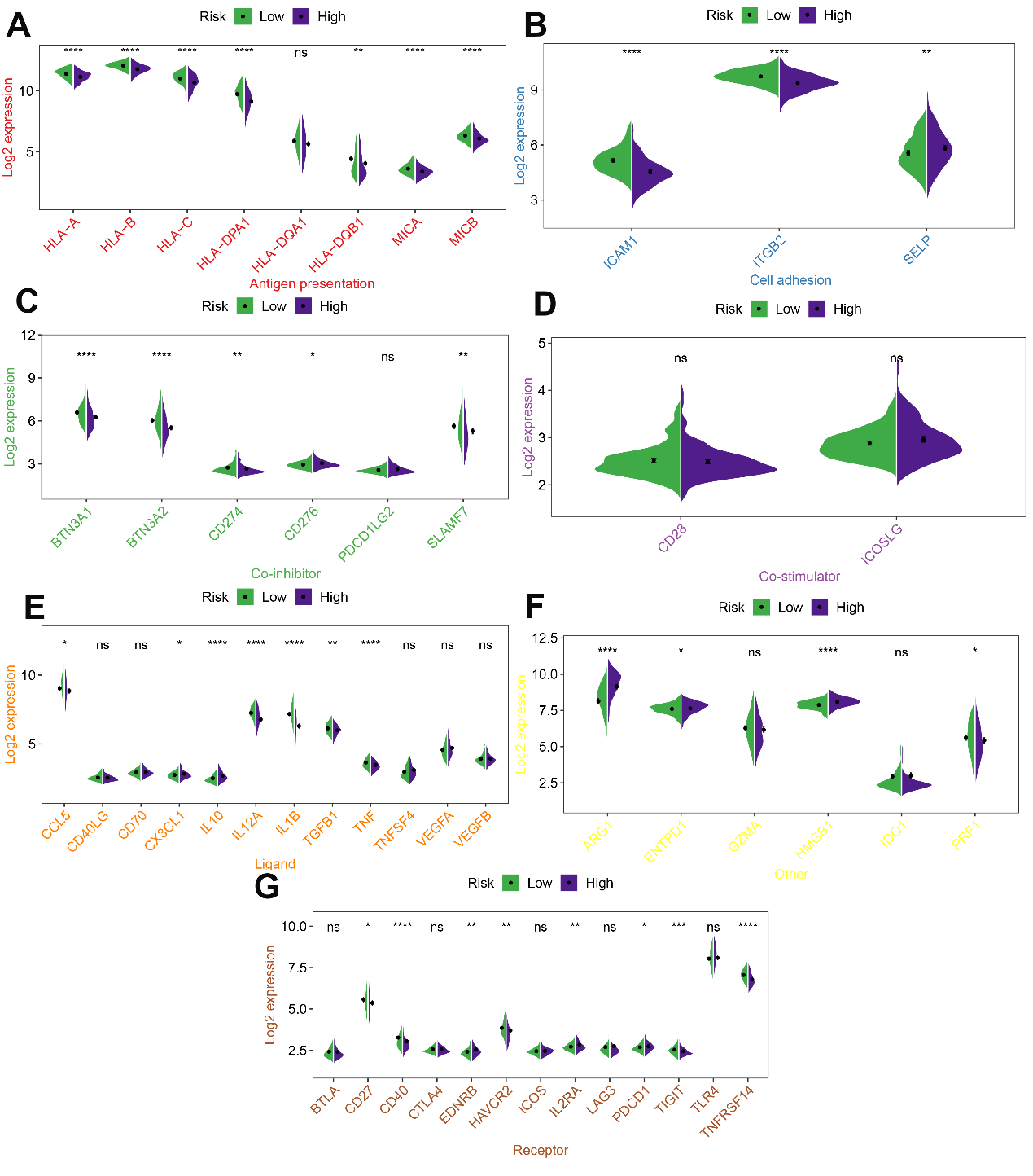


**Supplementary Figure 5**

**A-E.** Immune genes associated with antigen presentation, cell adhesion, co-inhibitor, ligand, receptor and other immune modulators in patients with different riskScore.


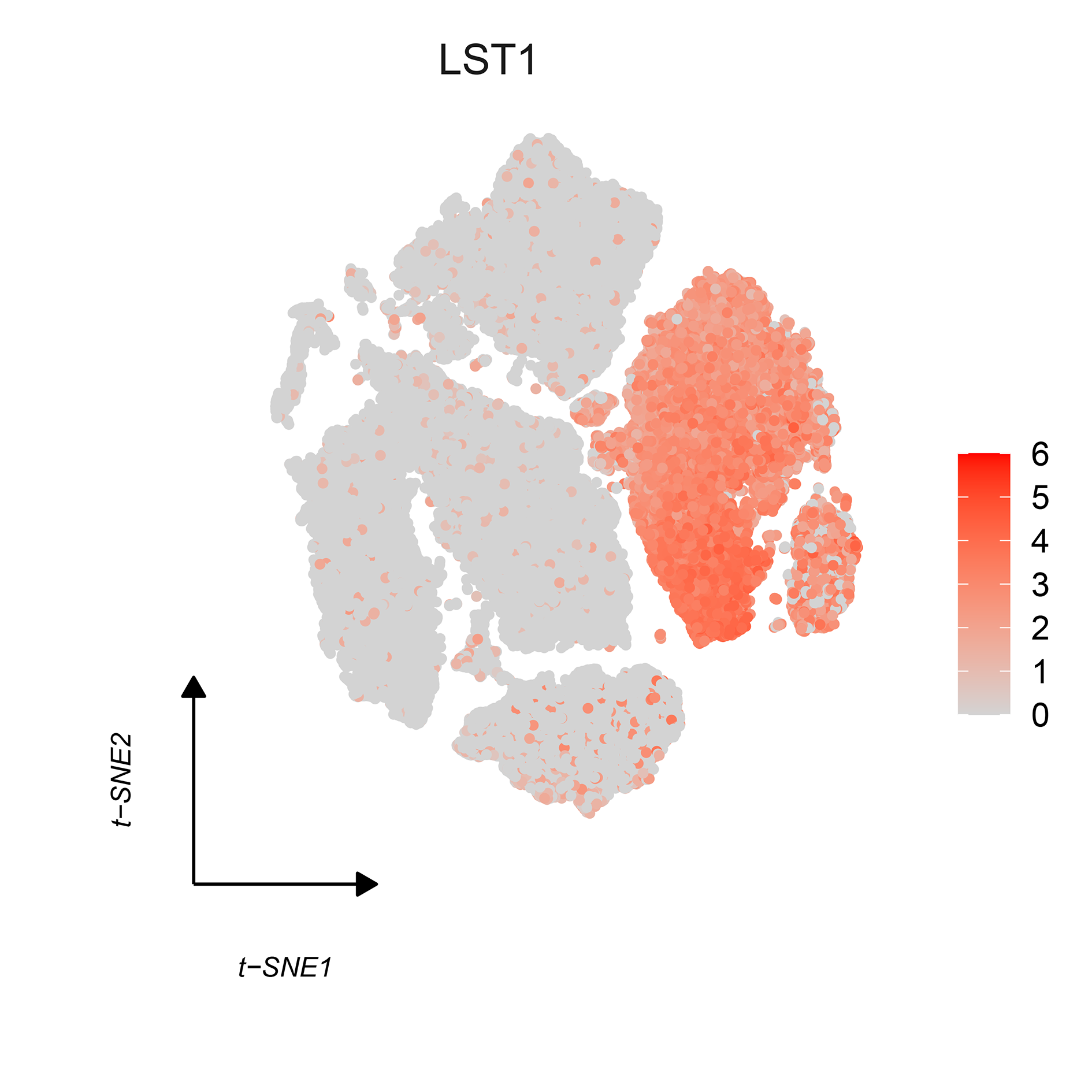


**Supplementary Figure 6**

The t-SNE plot showed the expression levels of LST1 in each celltype of single-cell samples.
